# Results of a phase Ib study of SB-121, an investigational probiotic formulation, a randomized controlled trial in participants with autism spectrum disorder

**DOI:** 10.1038/s41598-023-30909-0

**Published:** 2023-03-30

**Authors:** Lauren M. Schmitt, Elizabeth G. Smith, Ernest V. Pedapati, Paul S. Horn, Meredith Will, Martine Lamy, Lillian Barber, Joe Trebley, Kevin Meyer, Mark Heiman, Korbin H. J. West, Phoevos Hughes, Sanjeev Ahuja, Craig A. Erickson

**Affiliations:** 1 Division of Behavioral Medicine and Clinical Psychology, Cincinnati Children’s Hospital Medical Center, Cincinnati, OH USA; 2 Department of Pediatrics, University of Cincinnati College of Medicine, Cincinnati, OH USA; 3 Division of Child and Adolescent Psychiatry, Cincinnati Children’s Hospital Medical Center, Cincinnati, OH USA; 4 Division of Neurology, Cincinnati Children’s Hospital Medical Center, Cincinnati, OH USA; 5 Department of Psychiatry, University of Cincinnati College of Medicine, Cincinnati, OH USA; 6 Scioto Biosciences, Inc., Indianapolis, IN USA

**Keywords:** Drug discovery, Biologics, Medical research, Drug development

## Abstract

Autism Spectrum Disorder (ASD) is a neurodevelopmental disorder characterized by core impairments in social communication as well as restricted, repetitive patterns of behavior and/or interests. Individuals with ASD, which includes about 2% of the US population, have challenges with activities of daily living and suffer from comorbid medical and mental health concerns. There are no drugs indicated for the core impairments of ASD. As such, there is a significant need for the development of new medication strategies for individuals with ASD. This first-in-human placebo-controlled, double-blind, crossover study investigated the safety (primary objective) and efficacy of oral SB-121, a combination of L. reuteri, Sephadex® (dextran microparticles), and maltose administered once daily for 28 days in 15 autistic participants. SB-121 was safe and well tolerated. SB-121-associated directional improvements in adaptive behavior measured by Vineland-3 and social preference as measured with eye tracking were noted. These results provide support for further clinical evaluation of SB-121 as a treatment in autistic patients. To evaluate the safety and tolerability of multiple doses of SB-121 in subjects with autism spectrum disorder. Single-center, randomized, placebo-controlled, double-blind, crossover trial. 15 patients with autism spectrum disorder were randomized and analyzed. Daily dosing of SB-121 or placebo for 28 days, followed by approximately a 14 day washout, then 28 days of dosing with other treatment. Incidence and severity of adverse events, presence of *Limosilactobacillus reuteri* and Sephadex® in stool, and incidence of bacteremia with positive *L. reuteri* identification. Additional outcomes include changes from baseline on cognitive and behavior tests as well as biomarker levels. Adverse event rates were similar between SB-121 and placebo, with most reported as mild. There were no severe or serious adverse events. No participants had features of suspected bacteremia or notable changes in vital signs, safety laboratory, or ECG parameters from baseline. There was a statistically significant increase from baseline in the Vineland-3 Adaptive Behavior Composite score (p = 0.03) during SB-121 treatment. There was a trend for increased social/geometric viewing ratio following SB-121 treatment compared to placebo. SB-121 was safe and well tolerated. SB-121-associated directional improvements in adaptive behavior measured by Vineland-3 and social preference as measured with eye tracking were noted.

**Trial registration:** clinicaltrials.gov Identifier: NCT04944901.

## Introduction

Autism Spectrum Disorder (ASD) is a neurodevelopmental disorder characterized by core impairments in social communication and interaction combined with restricted, repetitive patterns of behavior and/or interests^1^. It is estimated that 1.7–2.8% of people of all ages are diagnosed with ASD in the United States^2,3^. Individuals with autism may struggle to function at school, work, and in everyday life situations. These challenges may be compounded by high rates of comorbid mental and physical health conditions^4–7^. These include, but are not limited to, gastrointestinal (GI), immunological, and psychiatric disorders. Overall, autistic individuals are at a 3 to 10 times higher risk for premature mortality^8,9^ compared to the general population. These issues highlight the critical need for the development of treatment options in ASD^10^.

Despite decades of research focused on the development of therapeutics for the treatment of the core social, communication, or functional impairments associated with autism, no such drugs have been approved by the United States Food and Drug Administration (FDA)^11^. The only approved drugs in ASD are aripiprazole and risperidone, which are limited to the treatment of irritability associated with physical aggression, self-injurious behavior, and severe tantrums in autistic youth^12^.

Enhancement of oxytocin signaling is among the more promising and well-studied targets of core symptom treatment development in ASD^11^. Oxytocin is an endogenous neuroendocrine hormone produced in the hypothalamus, released by the posterior pituitary into blood and stimulates milk letdown and uterine contractions in females. Hypothalamic oxytocin neurons also project to areas within the central nervous system (CNS) that are responsible for regulating social behavior^13–15^. Central oxytocin pathways also may project to the efferent vagal nervous system. In rats, intracisternal oxytocin administration reduces colonic hyperpermeability via the vagal cholinergic pathway^16^. The relationship between oxytocin activity and gastrointestinal symptomatology has not been directly explored in ASD.

Several studies have specifically investigated the oxytocin system in autism. A meta-analysis of studies using plasma oxytocin as a biomarker in ASD noted that many, but not all, autistic youth showed reduced oxytocin levels^17^. A meta-analysis of oxytocin receptor gene single-nucleotide polymorphisms noted an association between autism and certain polymorphisms^18^. However, exogenously administered oxytocin does not cross the blood–brain barrier. While this may potentially be overcome by the intranasal administration of oxytocin, clinical trials of intranasal administration of oxytocin in ASD have demonstrated mixed results^11^. Improvements in emotion recognition and social behavior were noted in several early phase trials^19–22^, while a large 24-week double-blind placebo-controlled parallel group trial of intranasal oxytocin in 290 autistic youth noted no treatment-associated positive clinical effects^23^. Study authors hypothesized that intranasal oxytocin administration may not adequately mimic the endogenous pulsatile oxytocin pattern and stimulation of the CNS oxytocin receptor, which may have contributed to lack of efficacy in this trial^24,25^. Given the importance of oxytocin in regulating social behavior and the challenges with exogenous administration, there is a clear need to evaluate alternative approaches to enhancing endogenous neuronal secretion of oxytocin in autistic individuals.

Studies show a high prevalence of gastrointestinal symptoms in patients with ASD, with autistic youth almost eight times more likely to suffer from significant gastrointestinal symptoms such as constipation, GI pain, or diarrhea than those with typical development^26^. Additionally, gastrointestinal symptoms in autistic patients have been demonstrated to correlate with the degree of maladaptive behavior such as irritability, social withdrawal, hyperactivity, and interfering repetitive behavior^26^.

*Limosilactobacillus reuteri* (*L. reuteri*) [Lr]*,* formerly known as *Lactobacillus reuteri*, is a probiotic bacterium that naturally colonizes the outer mucous layer of the intestines. *L. reuteri* stimulates production of mucin by goblet cells and protects intestinal cells from opportunistic pathogens. Oral *L. reuteri* treatment has been associated with reduction in social deficits in three mouse models of ASD through modulation of the gut-brain axis^27^. Although further studies are still on-going to precisely characterize this interaction, *L. reuteri* has been shown to stimulate the afferent vagus nerve to induce CNS oxytocin signaling^27^. It is hypothesized that these improvements have been driven by the ability of *L. reuteri* to stimulate oxytocin signaling to the ventral tegmental area of the CNS, a region with a significant role in reward, motivation, cognition, and aversion^27–30^.

SB-121 is a formulation of *L. reuteri* with Sephadex® (dextran microparticles, [DM]) and maltose. This combination results in a series of beneficial changes in the bacterium, including increased adherence to intestinal epithelial cells, improved gastric survival, and enhanced persistence through biofilm formation^31^. In this activated state, *L. reuteri* use has been associated with reduced disease incidence and severity, reduced intestinal inflammation and permeability, and reduced mortality in animal models of necrotizing enterocolitis (NEC) or *Clostridioides difficile* infection^32–34^.

Given the need to evaluate therapeutic methods to safely boost endogenous CNS oxytocin signaling in patients with ASD, combined with the clear high rates of gastrointestinal dysregulation in individuals with autism, we proposed to evaluate the safety of oral administration of SB-121 in adolescents and adults with autism. Secondarily, we proposed to evaluate the potential efficacy of SB-121 in ASD.

## Methods

We conducted a randomized double-blind, placebo-controlled crossover trial of SB-121 in fifteen 15–45 year old autistic participants. The sample size was not determined based on statistical assumptions. Evaluation of 15 total subjects was considered sufficient to allow evaluation of the study’s objectives. This was a double-blind study. The study team and subjects were blinded to the randomized study treatment assignments. In order to maintain the blind throughout the duration of the clinical study, all investigational product was affixed with a blinded label. Only the dispensing pharmacist was aware of study drug assignment. Randomization.com was utilized to generate the random allocation sequence, with two blocks of 8 patients per block. A randomization list was generated containing treatment assignments, and participants were added sequentially to the list following enrollment, and the corresponding study drug was dispensed. The study pharmacist generated the random allocation sequence, the study Principal Investigator enrolled participants, and the CCHMC Investigational Drug Service pharmacists assigned the participants. Participants were randomized in a 1:1 ratio to receive treatment with either SB-121 or placebo for 28 days. Following an approximately 14-day washout period, all participants crossed over to the other treatment (SB-121 or placebo) for 28 days.

This study was conducted at the Cincinnati Children’s Hospital and in accordance with ICH GCP, the United States (US) Code of Federal Regulations (CFR) and Cincinnati Children’s Hospital IRB. The protocol was reviewed and approved by the Cincinnati Children’s Hospital Institutional Review Board and registered at clinicaltrials.gov (NCT04944901) on 30/06/2021. All participants under age 18 or over 18 with a legal guardian had a parent or legally authorized caregiver provided informed consent for their participation. Each participant provided their own additional consent or assent as possible and applicable. All participant data have been de-identified in this work. All the inclusion and exclusion criteria are listed in Supplementary Information (Supplementary Table S1). In short, all participants had a confirmation of DSM-5 criteria-based ASD diagnosis using the Autism Diagnostic Observation Schedule, 2nd Edition (ADOS-2)^35^. Participants had to be free from active, uncontrolled GI symptoms or fever, and autoimmune disorders. Additional exclusion criteria included use of proton pump inhibitors, antibiotics, monoclonal antibodies, immunosuppressive drugs, and probiotics excluding yogurt; participants could maintain other medications and diet throughout study as long as they were stable. SB-121 (or placebo) was given daily and each dose consisted of 2 × 10^10^ colony forming units of *L. reuteri*, 200 mg of Sephadex®, and 74 mM of maltose in a final volume of 10.8 mL. Placebo consisted of 200 mg Sephadex® and 74 mM of maltose in a final volume of 10.8 mL. After reconstitution of either SB-121 or placebo, the mixture was left for 15–45 min at room temperature and then consumed mixed with a preferred drink (water or juice).

Following consent, a screening visit was completed including administration of the ADOS-2 (which was not required if assessment was completed within the previous 36 months and results were available), clinical interview using DSM-5 criteria for ASD, a medical and psychiatric history, physical examination and laboratory tests done to confirm study eligibility. Following randomization, but before the initial dose of SB-121 or placebo, additional subject characterization measures were completed including cognitive testing using the Wechsler Abbreviated Scale of Intelligence, 2nd Edition (WASI-II) and administration of the Social Communication Questionnaire (SCQ). In each SB-121 or placebo treatment period, participants or their caregivers completed additional assessments both pre-dose and following 28 days of daily dosing (i.e., at outcome) including the Vineland Adaptive Behavior Scales, 3rd edition (Vineland-3, Comprehensive Interview)^36^, Aberrant Behavior Checklist (ABC)^37^, Clinical Global Impressions Severity (CGI-S) and Improvement (CGI-I; done post-treatment only) subscales^38^, Woodcock Johnson 3rd Edition (WJ-III)^39^, Repeatable battery for assessment of neuropsychological status (RBANS)^40^, Test of attentional performance for children (KiTap)^41^, and Neurophysiology measures^42,43^. All report measures were conducted with the primary caregiver. The Vineland-3 was conducted by trained research staff and the CGI-S/CGI-I was conducted by a study physician. All study personnel were blinded to condition. Additionally, we evaluated quantitative subject performance using a social versus non-social scene preference eye tracking task^44^. Samples were collected for the evaluation of plasma oxytocin, plasma vasopressin, serum hs-CRP, tumor necrosis factor-α, stool lactoferrin, stool calprotectin and presence of Sephadex® microspheres in the stool at baseline and following 28 days of treatment with SB-121 and placebo (i.e., at the start and end of each period for four total collections). A 2-week washout period occurred between each treatment period. Last, any incidence of symptomatic bacteremia with positive *L. reuteri* identification was recorded. For a complete schedule of events, see Supplementary Table S2.

Regarding safety evaluations, participants completed safety laboratory panels (hematology and blood chemistry studies) and vital signs pre- and post-28 days of treatment during each treatment period. A full physical examination was done at screening and subsequently a limited focused physical examination was done for the evaluation of adverse events, as needed at all in person visits. Adverse events, concomitant medications and treatment compliance were assessed during all visits. All treatment emergent adverse effects (TEAEs) were recorded and tabulated for comparison across SB-121 or placebo treatment, as were the vital signs and the hematology and blood chemistry parameters.

We conducted analysis of change from baseline in the Vineland-3 composite and domain scores, ABC subscale scores, and CGI-S utilizing a general linear model where the change score (i.e., the difference of post-28 days of treatment value from the pre-dosing value for each of the two periods) served as dependent variable. The difference in score was modeled as a function of treatment (SB-121 or placebo), study period (1 or 2), and the sequence of treatments (SB-121 in the first period or second). Subject was included in the model as a random effect and the sequence term measured potential crossover effect. If no crossover effect was noted for an outcome measure, then the adjusted (least squared) means for the treatments were given along with their difference and a p-value was assigned for the null hypothesis of no difference. Given the pilot nature of this analysis, p-values were not corrected for multiplicity. Given that the CGI-I is a Likert scale rating of improvement and is not administered at pre-treatment/baseline, CGI-I mean values post-treatment were compared between SB-121 and placebo.

To obtain eye tracking data, participants were seated in a quiet, dark room in front of a Tobii XL300 eye tracker at a distance of 60–65 cm from the eye tracker monitor. Each participant was presented with verbal instructions to look at the screen. The eye tracker was calibrated for each participant at the beginning of the session using the Tobii Studio “five-point calibration”. Successful calibration was ascertained via Tobii Studio’s automated validation procedure. A second attempt to calibrate was conducted if the participant did not successfully calibrate initially. Following calibration, participants completed a social interest paradigm, as previously published^44^, where three silent 20 s side-by-side videos were presented with a social scene on one half of the screen and a geometric (i.e., non-social) pattern video on the other half (see Supplemental Fig. S1 for image of paradigm). The side of the social scene video was pseudo-randomized and switched after each 20-s segment. Social scene preference ratio was calculated by dividing the time spent viewing the social scene videos by the total time spent viewing the social scene or geometric pattern videos. Thus, positive values indicate a “social preference” with more time spent looking at the social scene versus geometric pattern, whereas negative values indicate a “non-social preference” with more time looking at the geometric pattern.

Raw eye tracking data was exported from Tobii Studio and areas of interest (AOI) were created using MATLAB (version R2019a; The Mathworks, Inc., Natick, Massachusetts). The AOIs included the social scene or the geometric scene. The proportion of looking time was calculated by dividing the looking time to the AOI region by the total looking time to the geometric + social scenes. Proportion of valid looking was calculated by dividing the total looking time to anywhere on the screen divided by the total stimulus presentation time. Participants were excluded if they had less than 35% valid looking data across the videos^44–46^. A generalized linear model was conducted with ratio of social versus non-social viewing as the dependent variable. The statistical analysis for all analyses, except eye tracking, were conducted using SAS® version 9.4 (SAS Institute Inc., Cary, NC). All eye tracking models were completed with SPSS. Cohen’s d effect sizes were included when appropriate.

## Results

**Figure 1:**
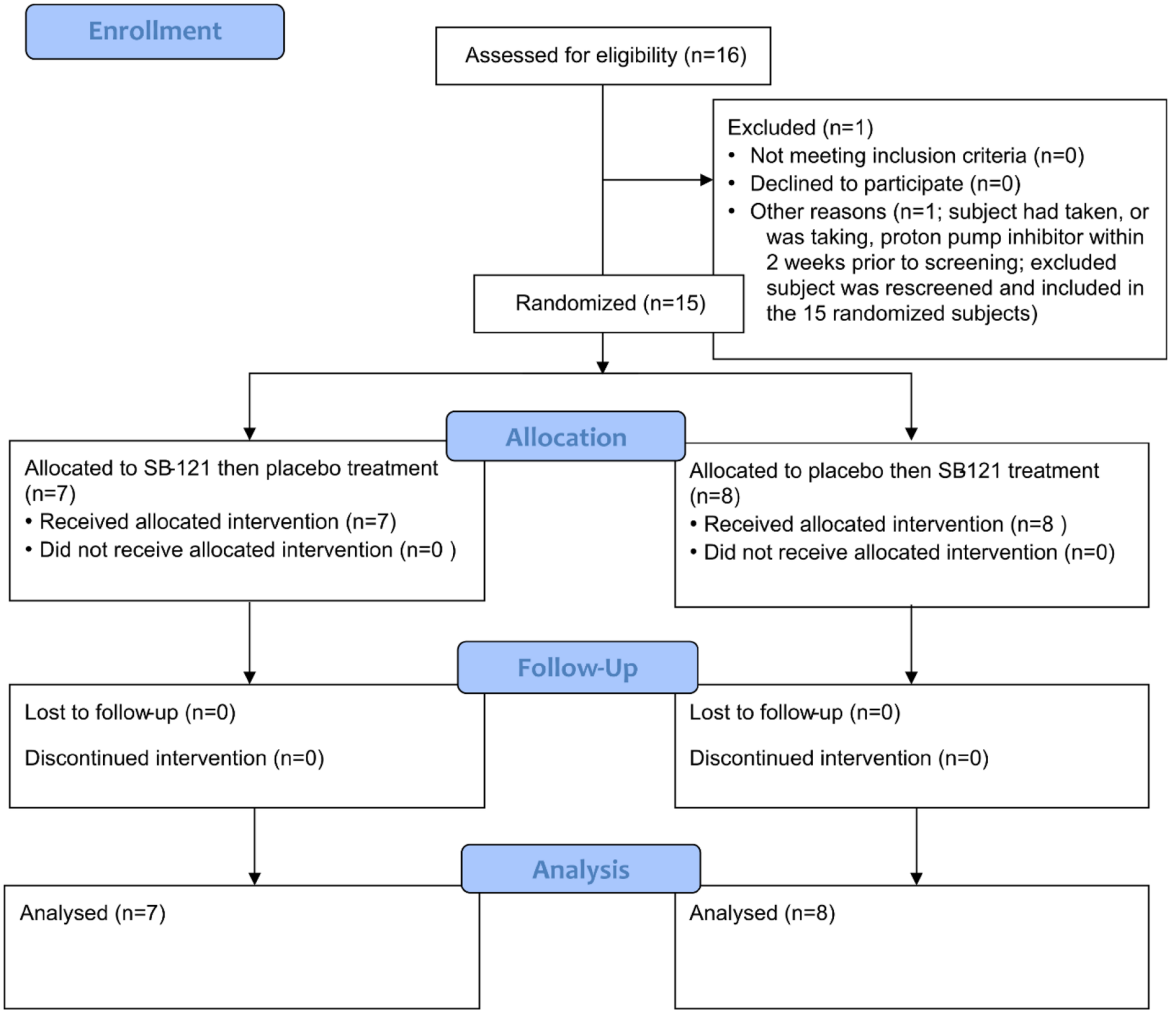
CONSORT diagram. The first participant was enrolled 02 AUG 2021. The last study visit occurred on 03 MAR 2022.

Sixteen screening visits were conducted involving 15 individual participants. One participant screen failed due to concomitant proton pump inhibitor use and was subsequently rescreened and eventually randomized; see CONSORT Diagram in Fig. 1 for study flow detail. Eight participants were randomized to receive placebo first and seven received SB-121 first. All randomized participants completed both treatment periods. No effects were observed due to treatment sequence. Despite the protocol being open to male and female participants, all enrolled participants were male ranging in age from 15 to 27 years. Please see Table 1 for additional participant demographic details.

**Table 1:** Summary of demographic and baseline characteristics (intent to treat population).

Characteristic statistic	SB-121 received first (N = 7)	Placebo received first (N = 8)	Total (N = 15)
Age (years)			
n	7	8	15
Mean (SD)	20.1(1.46)	19.9 (4.09)	20.0 (3.05)
Median	20.0	19.5	20.0
Min, max	18, 22	15, 27	15, 27
Sex, n (%)			
Male	7 (100.0)	8 (100.0)	15 (100.0)
Female	0	0	0
Race, n (%)			
White	7 (100.0)	7 (87.5)	14 (93.3)
Black or African American	0	0	0
Asian	0	1 (12.5)	1 (6.7)
Ethnicity, n (%)			
Not Hispanic or Latino	7 (100.0)	8 (100.0)	15 (100.0)
Height (cm) at Baseline			
n	7	8	15
Mean (SD)	177.96 (6.904)	178.54 (6.280)	178.27 (6.343)
Median	179.40	180.75	179.70
Min, Max	169.0, 189.3	170.3, 186.2	169.0, 189.3
Weight (kg) at baseline			
n	7	8	15
Mean (SD)	69.67 (9.725)	98.26 (40.006)	84.92 (32.538)
Median	69.00	91.15	75.80
Min, Max	59.0, 87.5	62.3, 188.9	59.0, 188.9
Body mass index (kg/m^2^) at baseline^a^			
n	7	8	15
Mean (SD)	22.11 (3.859)	31.29 (14.552)	27.01 (11.606)
Median	21.20	29.25	21.60
Min, Max	19.0, 30.6	18.0, 65.1	18.0, 65.1

*ADOS-2* Autism Diagnostic Observation Schedule, 2nd edition, *max* maximum, *min* minimum, *n* number of participants with data available, *N* number of participants according to the first treatment sequence, *SD* standard deviation, % percentages were calculated based on N as the denominator.

Baseline was considered the last observation prior to dosing in Treatment Period 1.

Drug abuse was considered positive if at least one of the parameters of drug abuse was positive and it was considered negative if all parameters of drug abuse were negative.

^a^Body mass index (kg/m^2^) = body weight (kg)/height (m^2^).

Baseline WASI-II full scale values for the 15 randomized participants in the study showed a mean full-scale IQ of 88.66 (SD 29.2; range 40–128). The SCQ, used as an index of core autism symptom severity, had a mean score of 20.2 (SD 8.06; range 4–34). WASI and SCQ scores did not significantly differ for participants based on randomization order. Overall, the wide variance in WASI-II and SCQ scores in this study sample is consistent with that seen in patients with ASD clinically. Two participants scored ≤ 8 on the SCQ, but based on ADOS-2 and consensus diagnosis still met criteria for ASD. Regarding concomitant medication use, 15 (100.0%) received at least one concomitant medicine during the study (see Supplementary Information Tables S3 and S4 for full concomitant medication use data). The most frequently reported (≥ 10% of participants) concomitant medications for both the Treatment Period 1 and Treatment Period 2 included melatonin (4 [26.7%] participants); acamprosate, buspirone, clonidine, dexmethylphenidate hydrochloride, lisdexamphetamine mesilate, quetiapine, sertraline, metformin, vitamin D (3 [20.0%] participants each); amphetamine aspartate/amphetamine sulfate/dexamphetamine saccharate/dexamphetamine sulfate, guanfacine hydrochloride, methylphenidate hydrochloride, risperidone, vitamins, and fish oil (2 [13.3%] participants each).

Overall, use of SB-121 was well tolerated. Mean treatment compliance was similar between both treatment periods and treatment assignment. For SB-121 these were 92.2% and 90.4% for Periods 1 and 2 respectively; for placebo 95.7% and 84.3%. The treatment compliance data indicates that that the reconstitution and dosing instructions for the study drug (SB-121, placebo) were not a barrier to compliance and that it was well tolerated.

Treatment emergent adverse event (TEAE) and treatment related TEAE rates were similar between SB-121 and placebo (Tables 2, 3). During treatment period 1, among 11 participants with at least one TEAE, 5 received SB-121 (71.4%) and 6 (75.0%) received placebo. During treatment period 2, of 5 participants with at least one TEAE, 2 (25.0%) received SB-121 and 3 (42.9%) received placebo. Overall, during SB-121 treatment, 7 of 15 participants (46.7%) reported a total of 14 TEAEs, with the most common being gastrointestinal TEAEs reported by 3 (20.0%) participants. On the placebo arm, 9 (60.0%) of participants reported 23 TEAEs, with the most common being gastrointestinal TEAEs reported by 4 (26.7%) participants. Most of the adverse events reported on either arm were mild. There were no severe or serious adverse events during the trial. No participants discontinued study treatment due to adverse events. No participants had features of suspected bacteremia during the study. No changes from baseline in vital signs and safety laboratory and ECG parameters were noted with placebo or SB-121 treatment.

**Table 2:** Treatment-emergent adverse events (TEAEs).

System organ class Preferred term	Treatment	
	SB-121 (N = 15) n (%)	Placebo (N = 15) n (%)
Number of Subjects with at least one TEAE	7 (46.7)	9 (60.0)
Blood and lymphatic system disorders	1 (6.7)	0
Leukocytosis	1 (6.7)	0
Gastrointestinal disorders	3 (20.0)	4 (26.7)
Diarrhea	2 (13.3)	3 (20.0)
Abdominal pain	0	1 (6.7)
Abdominal pain upper	1 (6.7)	0
Nausea	1 (6.7)	0
Vomiting	0	2 (13.3)
General disorders and administration site conditions	1 (6.7)	1 (6.7)
Fatigue	1 (6.7)	0
Pain	0	1 (6.7)
Infections and infestations	2 (13.3)	3 (20.0)
Sinusitis	2 (13.3)	0
COVID-19	0	1 (6.7)
Tinea infection	0	1 (6.7)
Upper respiratory tract infection	0	1 (6.7)
Investigations	0	1 (6.7)
Alanine aminotransferase increased	0	1 (6.7)
Nervous system disorders	1 (6.7)	2 (13.3)
Headache	1 (6.7)	2 (13.3)
Psychiatric disorders	0	1 (6.7)
Anxiety	0	1 (6.7)
Depression	0	1 (6.7)
Insomnia	0	1 (6.7)
Renal and urinary disorders	0	1 (6.7)
Bilirubinuria	0	1 (6.7)
Respiratory, thoracic and mediastinal disorders	2 (13.3)	2 (13.3)
Cough	2 (13.3)	0
Nasal congestion	0	1 (6.7)
Oropharyngeal pain	0	1 (6.7)

**Table 3:** Treatment related TEAEs.

System organ class Preferred term	Treatment	
	SB-121 (N = 15) n (%)	Placebo (N = 15) n (%)
Number of Subjects with at least one study treatment related TEAE	3 (20.0)	4 (26.7)
Blood and lymphatic system disorders	1 (6.7)	0
Leukocytosis	1 (6.7)	0
Gastrointestinal disorders	3 (20.0)	4 (26.7)
Diarrhea	2 (13.3)	3 (20.0)
Abdominal pain	0	1 (6.7)
Abdominal pain upper	1 (6.7)	0
Vomiting	0	1 (6.7)

**Figure 2:**
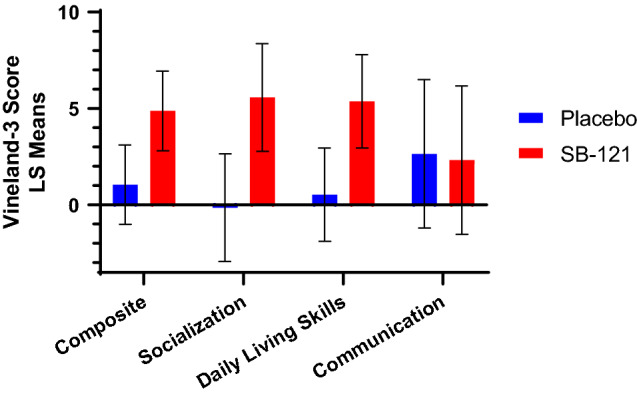
Vineland-3 change from baseline scores (LS means, SEM).

Regarding our analysis of outcome measures in evaluating potential clinical response to SB-121 treatment in these patients with autism, SB-121 treatment was associated with improvement in Vineland-3 scores compared to placebo in the entire subject sample (see Fig. 2, Table 4). The Vineland-3 Adaptive Behavior Composite Score change from baseline (LS Means) in the SB-121 arm was statistically significant (p = 0.03), and specifically within the Vineland-3 Daily Living Skills Domain (p = 0.04). Additionally, there were small but positive SB-121 associated treatment effect size estimates of 0.32–0.41 across the Adaptive Behavior Composite Score, and the Socialization and Daily Living Skills domains.

**Table 4:** Summary of Vineland-3 Scores (change from baseline).

Measure	Placebo			SB-121			SB-121 minus placebo			Approximate treatment effect size
	LS mean	SEM	P-value	LS mean	SEM	P-value	LS mean	SEM	P-value	
Vineland adaptive behavior composite score	1.05	2.06	0.61	4.87	2.06	0.03	3.81	3.21	0.26	0.32
Vineland socialization domain	− 0.14	2.79	0.96	5.57	2.79	0.06	5.71	4.81	0.26	0.32
Vineland daily living skills domain	0.53	2.42	0.83	5.37	2.42	0.04	4.84	3.14	0.15	0.41
Vineland communication domain	2.65	3.85	0.50	2.32	3.85	0.55	− 0.33	5.74	0.96	− 0.02

*LS* least squares, *SEM* standard error of the mean.

For each measurement, the response was the difference between post-dose and pre-dose value. The adjusted, or LS mean for each treatment (SB-121 or placebo) and its standard error were derived from the model. The LS means differences between SB-121 and placebo (SB-121 LS mean minus Placebo LS mean) and their standard errors were also assessed. Statistical significance was set at a two-sided alpha = 0.05 and no adjustment was made for multiple comparisons. An approximate treatment effect was derived from the difference between the LS means for the SB-121 and placebo change from baseline and its standard error. All statistical analyses were conducted using SAS® statistical software version 9.4 for Windows (SAS Institute Inc., Cary, NC).

**Figure 3:**
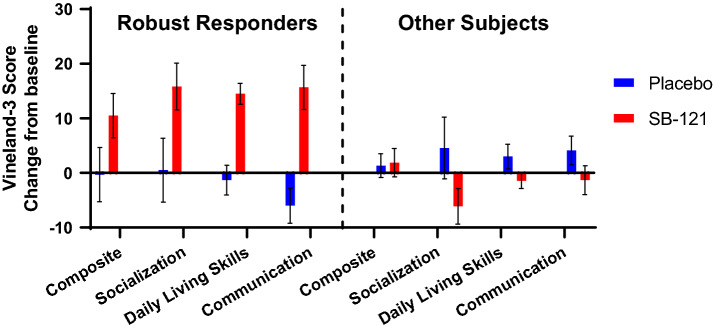
Vineland-3 change from baseline scores—robust responders and other subjects (mean, SEM).

Given the variable phenotypic presentation of our sample (based on WASI and SCQ), we examined individual-level responses and noted six participants had a robust Vineland-3 response indicated by an Adaptive Behavior Composite score change from baseline while on SB-121 versus placebo of ≥ 8 (Fig. 3, Table 5, Supplementary Information Table S5). However, follow-up analyses comparing these so-called “robust responders” and “other subjects” on clinical measures or oxytocin levels at baseline did not reveal any significant differences. Considering improvement in this parameter by ≥ 8 compared to placebo as a robust response is a conservative estimate driven by prior clinical trial use of the Vineland in ASD where subject samples have noted Vineland Adaptive Behavior Composite score standard deviations ranging from ~ 6 to ~ 16^47,48^ indicating a ≥ 8 response would clinically represent in theory effect sizes from ~ 0.5 to ~ 1.25 in a responding population of subjects.

**Table 5:** Vineland-3 robust responders and other subjects (change from baseline).

	Robust responders n = 6				Other subjects n = 9			
	Placebo		SB-121		Placebo		SB-121	
	Mean	SEM	Mean	SEM	Mean	SEM	Mean	SEM
Vineland adaptive behavior composite score	− 0.33	4.97	10.50	4.06	1.33	2.18	1.89	2.60
Vineland socialization domain	0.50	5.86	15.83	4.29	4.56	5.64	− 6.11	3.27
Vineland daily living skills domain	− 1.33	2.73	14.50	1.91	3.00	2.25	− 1.44	1.40
Vineland communication domain	− 6.00	3.21	15.67	4.06	4.11	2.64	− 1.33	2.64

*SEM* standard error of the mean.

Findings from the Aberrant Behavior Checklist (ABC) are presented in Table 6. The mean pre-treatment baseline raw scores across all ABC subscales were < 10 across both treatment periods. Mean baseline scores for the Irritability and Stereotypic Behavior subscales were in the 3–4 range. These baseline findings could denote limited interfering behavioral challenges in this cohort of study participants. No SB-121-associated significant or directional changes were noted across all subscales of the ABC.

**Table 6:** Pre- and post-dose data for ABC.

	Placebo				SB-121			
	Pre-dose		Post-dose		Pre-dose		Post-dose	
	Mean	SEM	Mean	SEM	Mean	SEM	Mean	SEM
Aberrant behavior checklist irritability subscale	3.13	1.01	2.4	0.9	4	1.18	3.13	1.09
Aberrant behavior checklist social withdrawal subscale	8.47	2.23	5.67	1.77	9.2	2.24	7.4	1.94
Aberrant behavior checklist stereotypic behavior subscale	3.2	0.87	1.93	0.77	3.33	0.91	3.27	0.83
Aberrant behavior checklist hyperactivity subscale	8.07	1.89	6.07	1.52	7.40	1.57	7.67	1.54
Aberrant behavior checklist inappropriate speech subscale	2.67	0.66	2.33	0.67	2.73	0.62	2.53	0.68

*ABC* Aberrant Behavior Checklist, *SEM* standard error of the mean.

Regarding subscales of the CGI, with the CGI-S there were no relevant differences between placebo and SB-121 and the results suggested stable scores during SB-121 and placebo treatment. Mean CGI-I scores were also similar post-SB-121 versus placebo treatment (see Supplementary Information Table S6).

**Figure 4:**
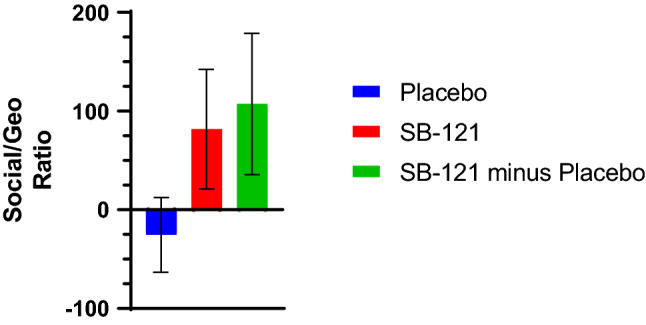
Eye tracking change from baseline score—social/geometric ratio (LS means, SEM).

**Figure 5:**
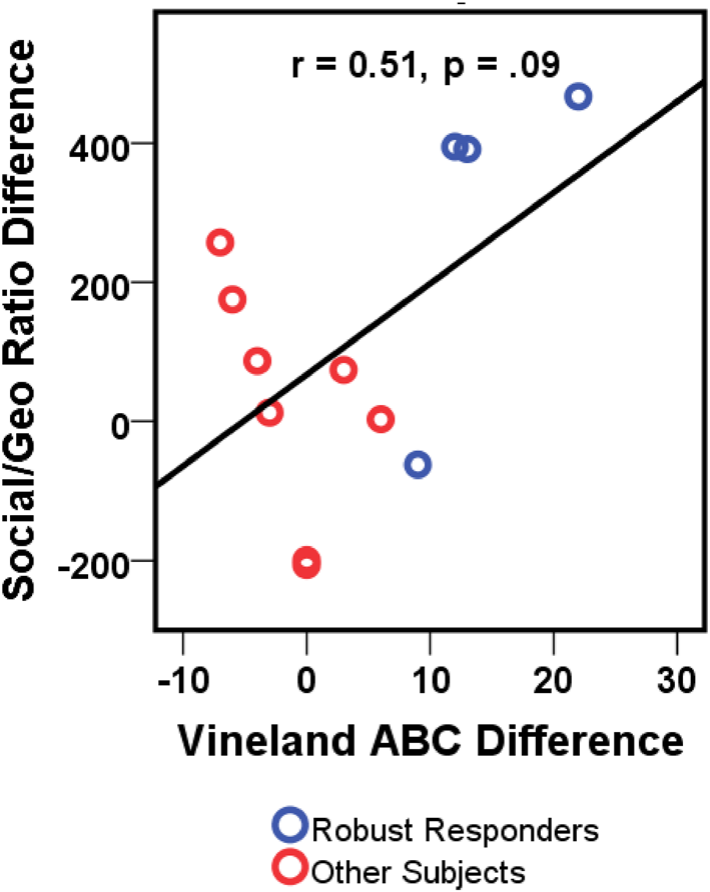
Relationship between Vineland-3 change from baseline and social/geometric ratio change from baseline following SB-121.

Utilizing eye tracking, there was a trend for increased social/geometric viewing ratio following SB-121 treatment compared to placebo (Fig. 4), such that more time was spent looking at the social compared to geometric scene (p = 0.1; SB-121 minus placebo of 107:1). This had a medium effect size of 0.61. When participants received SB-121 their ratio of viewing social versus non-social scenes was over 80:1, whereas viewing of non-social scenes versus social scenes was over 25:1 during placebo treatment. This suggests post-SB-121 treatment there was an increased social versus non-social preference during this task. Of note, there was a trending relationship between increased scores on the Vineland-3 Adaptive Behavior Composite and increased social scene viewing preference following SB-121 (Fig. 5; r = 0.51, p = 0.09).

**Figure 6:**
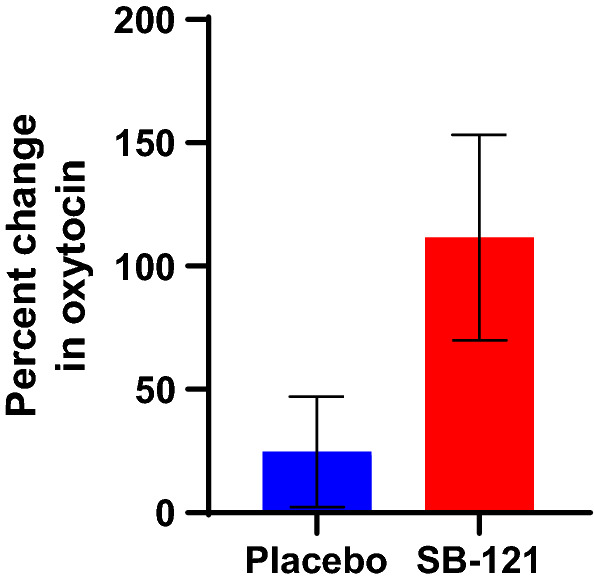
Plasma oxytocin percent change from baseline values (mean, SEM).

The mean (SD) percentage changes from baseline to Day 28 in plasma oxytocin levels for SB-121 and placebo groups were 111.63% (155.93) and 24.67% (80.94), respectively (Fig. 6, Supplementary Information Table S7). The p-value for this difference is based on a paired t-test, p = 0.106, where the unpaired SB-121 is omitted from the calculation. However, percent change in oxytocin levels did not relate to changes in Vineland-3, ABC, or social viewing ratio (p’s > 0.05).

No SB-121-associated directional or significant changes were noted across all subscales of the WJ-III, RBANS, KiTap or Neurophysiology measures. Additionally, there were no relevant changes in the biomarkers tested for plasma vasopressin, TNF-α, and HS-CRP, and stool calprotectin and lactoferrin. Assessment of stool samples indicated near to complete clearance of Sephadex® microspheres following treatment discontinuation. None of the subjects of either treatment groups showed any clinical features of suspected bacteremia during the study.

## Discussion

Overall SB-121 use was well tolerated and safe in 15 adolescents and adults with ASD. Treatment compliance in this pilot study was excellent which indicated that, given a standardized approach to reconstituting the formulation, patients would be able to take it as instructed. The enrolled participant sample was well representative of males with ASD broadly marked by variation in cognitive skills and severity of core ASD symptoms at baseline. Clinically, we documented clear directional improvements in adaptive behavior as measured by Vineland-3, which warrants replication in larger-scale study. Given the broad inclusion criteria for ASD utilized in this first-in-humans study, the Vineland-3 results are of particular interest as adaptive behavior deficits would be broadly expected in participants with ASD and this may have enabled this outcome measure to detect change in the context of significant baseline sample phenotypic heterogeneity. Additionally, the use of broader inclusion criteria in this study has allowed us to observe differences in response to SB-121, as some participants responded more robustly to treatment than others. Studying a diverse group of participants with ASD may help identify the subpopulations that respond more readily to treatment.

The Vineland-3 findings are in contrast to behavioral findings from the ABC where no trends in SB-121-associated change were noted. This may be due to a lack of impact of SB-121 on interfering behaviors in ASD, but also may be due to significant likely floor effects with use of the ABC in this specific sample of autistic individuals who had very low ABC scores at baseline. Broad interpretation of ABC score change in ASD trials is difficult in cases where inclusion criteria do not pre-specify ABC subscale or even an ABC total threshold score for study inclusion. It is possible that an early phase ASD clinical trial recruitment may bias towards enrollment of participants without significant interfering behavior that could preclude active study participation over a series of in person trial visits.

Although treatment-associated directional improvement was noted on the Vineland-3, this improvement was not simultaneously captured by clinician CGI-I ratings. The CGI-I is a clinician-rated global measure that takes into account all information available including interview with subject and primary caregiver; however, in the current study, daily functional skills were not specifically asked about and thus would not have been incorporated into the CGI-I rating unless the primary caregiver shared this information with the clinician. In addition, the CGI-I may be more prone to placebo effect especially in the context of a small first-in-disorder trial since reporting may tend to more uniformly positive than detailed, item by item, report. Last, it is also possible that the potential disconnect between VABS-3 improvement and CGI-I rating is a product of small sample size statistical modeling.

The finding that SB-121 was associated with a trend towards increased preference for viewing social versus non-social stimuli represents a quantitative, performance-based confirmation that this treatment could potentially enhance social interest in autistic individuals. Importantly, this was a trending quantitative correlate to improved adaptive behavior noted with treatment suggesting improved adaptive behavior as rated by a parent-rated measure may generalize to more real-world performance, especially within social interest. Thus, our findings warrant a larger-scale study for replication and extension.

Additional limitations of this report include a very small sample size that limited any ability to phenotypically define the subgroup of participants who appear to drive the overall positive directional findings of Vineland-3, ABC score, and social viewing as measured by eye tracking. To address this potential disconnect between CGI-I and VABS-3 ratings, in future study consideration to including prompts to evaluate daily functioning more thoroughly during the clinical CGI-I evaluation may be warranted. Further, since females were not represented in this study this omission is a study weakness and is likely a result of a small sample size and not stratifying enrollment by sex assigned at birth. Given the Phase I nature of the pilot study, had the sample included female participants, we would have not had any power to detect potential sex-associated differences in SB-121 tolerability or clinical response. Despite the gastrointestinal focus of the SB-121 intervention, we lack detailed assessment of GI symptoms in this trial, including potential quantitative change of stool features following SB-121 treatment. In future work it will be important to quantitatively evaluate GI symptoms at baseline and following treatment including potential use of quantitative evaluation of stool sampling given SB-121 direct exposure is limited to the gastrointestinal lumen. While a positive trend for plasma oxytocin measurements was observed when participants were taking SB-121, we note that there are significant limitations to plasma oxytocin measurements. Oxytocin is a notoriously difficult hormone to assay due to having a short half-life, being poorly immunogenic, and its tendency to bind to molecules in plasma^49^. Additionally, several participants had values below the lower limit of quantification at baseline, making it difficult to accurately quantify change with SB-121. Additionally, there are temporal differences between the release of central oxytocin and the accompanying secretion into the periphery; as such, plasma oxytocin levels should be treated with caution regarding its correlation with central oxytocin^50^.

This Phase Ib study report on the first-in-humans use of SB-121 in male autistic participants provides data supporting future larger placebo-controlled studies of this compound in this autistic patients. The findings of a favorable safety profile of SB-121 in this sample of 15 individuals combined with significant improvements in Adaptive Behavior with treatment that is further corroborated by performance-based positive change in social engagement, indicates this treatment may be of benefit in ASD. It will be important in larger-scale studies to make efforts to rigorously phenotype the medical and gastrointestinal profile of participants in addition to classic clinical descriptions of behavior and cognition to potential define a subgroup who may best respond to treatment. This is of particular importance given the potential strong subgroup response we noted even in this small-scale study that appeared to drive overall positive clinical change.

## Supplementary Information

Supplementary Information.

## Data Availability

Data is available on ClinicalTrials.gov (NCT049449) and can be requested from Scioto Biosciences (medinfo@sciotobiosciences.com).
