# Supplementary material for: Results of a phase Ib study of SB-121, an investigational probiotic formulation, a randomized controlled trial in participants with autism spectrum disorder
Source: Sci Rep. 2023 Mar 30;13:5192. doi: 10.1038/s41598-023-30909-0 (PMC10061375; doi:10.1038/s41598-023-30909-0)
Supplement: Supplementary file 1 — Supplementary Information. [file 41598_2023_30909_MOESM1_ESM.docx]

**Supplementary Information:**

**Results of a phase Ib study of SB-121, an investigational probiotic formulation, a randomized controlled trial in participants with autism spectrum disorder**

Lauren M. Schmitt^1,2^

Elizabeth G. Smith^1,2^

Ernest V. Pedapati^3,4,5^

Paul Horn^2,4^

Meredith Will^1,2^

Martine Lamy^3,5^

Lillian Barber^3,5^

Joe Trebley^6^

Kevin Meyer^6^

Mark Heiman^6^

Korbin H.J. West^6^

Phoevos Hughes^6^

Sanjeev Ahuja^6^

*Craig A. Erickson^3,5^

^1^Division of Behavioral Medicine and Clinical Psychology, Cincinnati Children’s Hospital Medical Center, Cincinnati, OH, United States.

^2^Department of Pediatrics, University of Cincinnati College of Medicine, Cincinnati, OH, United States.

^3^Division of Child and Adolescent Psychiatry, Cincinnati Children’s Hospital Medical Center, Cincinnati, OH, United States.

^4^Division of Neurology, Cincinnati Children’s Hospital Medical Center, Cincinnati, OH, United States.

^5^Department of Psychiatry, University of Cincinnati College of Medicine, Cincinnati, OH, United States.

^6^Scioto Biosciences, Inc.

*Corresponding author.

**Supplemental Figure 1: Social Versus Geometric Eye Tracking Paradigm.** Screenshot example from our side-by-side social versus geometric eye tracking paradigm. The red dots are qualitatively created by the Tobii Studio software as exemplars of gaze location with larger dots indicating longer gaze duration.


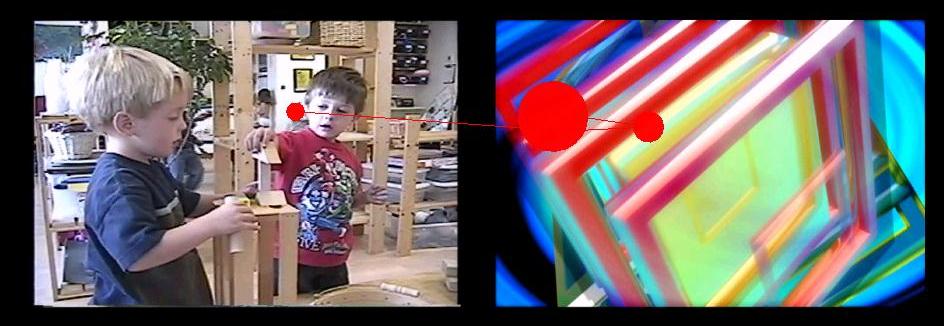


-

**Table S1: Inclusion and Exclusion Criteria.**

| **Inclusion Criteria** | Subject/parent (or authorized designee) had provided written informed consent for the study. |
| --- | --- |
|  | Subject was ≥15 and ≤45 years of age at the time of enrollment. |
|  | Diagnosis of AD as confirmed by the gold-standard clinical interview using Diagnostic and Statistical Manual of Mental Disorders, 5th edition (DSM-5 checklist) and administration of the Autism Diagnostic Observation Schedule, 2nd edition (ADOS 2). |
|  | Subject, if female and of childbearing potential, was not lactating or pregnant. |
|  | Subject, if female, was either not of childbearing potential or was practicing an acceptable effective method of birth control. |
|  | Subject was willing to comply with all study requirements (including the requirements for stool sampling and biobanking) and to return to the study facility for the follow up evaluations, as required. |
| **Exclusion Criteria** | Subject had known allergy or significant adverse reaction to Lr, Sephadex®, maltose, or related compounds. |
|  | Subject previously had GI surgery, intestinal obstruction, Clostridium difficile infection, or diverticulitis. |
|  | Subject had travelled outside of the USA in the 30 days prior to screening. |
|  | Subject had a diarrheal illness in 30 days prior to screening. |
|  | Subject had fever or active/uncontrolled GI symptoms (e.g., nausea, vomiting, diarrhea, constipation, abdominal distention, abdominal pain/cramps, flatulence) at the screening or had these within 14 days prior to screening. If the GI symptoms were stable, in the opinion of the investigator, the subject could have been enrolled. |
|  | Subject had any immunological/autoimmune disorder including, but not limited to, systemic lupus erythematosus, rheumatoid arthritis, Sjögren’s syndrome, inflammatory bowel disease, or immunoglobulin deficiency disorder, that would have increased the risk to the subject or interfered with the evaluation of SB-121. |
|  | Subject had a documented history of human immunodeficiency virus, hepatitis B, and/or hepatitis C. |
|  | Subject had implanted prosthetic devices including prosthetic heart valves. |
|  | Subject had implanted prosthetic devices including prosthetic heart valves. |
|  | Subject had taken, or was taking, any of the following prohibited medications:   - A proton pump inhibitor within 2 weeks prior to screening - Use of supplemental probiotics within 2 weeks prior to screening except for yogurt - Use of immunosuppressive medications, including corticosteroids at the screening - Treatment with monoclonal antibodies within 4 weeks prior to screening - Systemic antibiotics within 2 weeks prior to screening. |
|  | Subject had diabetes mellitus or was prediabetic. |
|  | Subject had received any IP (or investigational device) within 30 days prior to screening. |
|  | Subject had any of the following laboratory test results at screening:   - An absolute neutrophil count of <1.5 × 109/L - Alanine aminotransferase or aspartate aminotransferase >1.5 × upper limit normal (ULN), total bilirubin >1.5 × ULN (participants with known Gilbert’s Syndrome could have been included) - Serum creatinine >1.5 × ULN - Any other abnormal laboratory test that was clinically significant in the judgment of the investigator. |
|  | Subject had an unstable medical condition or was otherwise considered unreliable or incapable, in the opinion of the investigator, of complying with the requirements of the protocol. |
|  | Subject tested positive for drugs of abuse in a urine drug screen at screening. |
|  | Subject had a history of alcohol abuse. |

Abbreviations: DSM5 = Diagnostic and Statistical Manual of Mental Disorders, 5th edition; ADOS 2 = Autism Diagnostic Observation Schedule, 2nd edition; Lr *= Limosilactobacillus reuteri*; GI = gastrointestinal; IP = investigational product; ULN = upper normal limit.

**Table S2: Schedule of Events**

|  |  | **Study Period 1** | | | | | | **WASH OUT (14-21 Days)** | **Study Period 2** | | | | | | **Post-trial Washout** |
| --- | --- | --- | --- | --- | --- | --- | --- | --- | --- | --- | --- | --- | --- | --- | --- |
|  | **Day**  **-14 to 0** | **Day 1** | **Day 1** | **Day 7 (+/-2)** | **Day 14 (+/-2)** | **Day 21 (+/- 2)** | **Day 28 (+/-3)** | **Day 35 (+/-2)** | **Day 1^h^** | **Day 1** | **Day 7 (+/-2)** | **Day 14 (+/-2)** | **Day 21 (+/- 2)** | **Day 28 (+/-3)** | **Day 42 (+4)** |
| **Measure** | **Screen** | **Visit 1: Pre-dose** | **Visit 1: Post-dose** | **Follow- Up 1**  **Phone** | **Follow -Up 2**  **Phone** | **Follow- Up 3**  **Phone** | **Visit 2: Final Visit of Period 1** | **Wash out period call** | **Visit 3: Pre-dose** | **Visit 3: Post-dose** | **Follow Up 1**  **Phone** | **Follow Up 2**  **Phone** | **Follow Up 3 Phone** | **Visit 4: End-of-Study / Early Termination Visit** |  |
| **Informed Consent** | **X** |  |  |  |  |  |  |  |  |  |  |  |  |  |  |
| **Med/Psych Hx** | **X** |  |  |  |  |  |  |  |  |  |  |  |  |  |  |
| **Physical Exam^a^** | **X** |  |  |  |  |  | **X** |  | **X** |  |  |  |  | **X** |  |
| **Safety Labs^b^** | **X** |  |  |  |  |  | **X** |  | **X** |  |  |  |  | **X** |  |
| **Pregnancy Test (females)** | **X** | **X** |  |  |  |  | **X** |  | **X** |  |  |  |  | **X** |  |
| **Biomarker Blood Draw** | **X** |  |  |  |  |  | **X** |  | **X** |  |  |  |  | **X** |  |
| **Vital Signs** | **X** | **X** | **X** |  |  |  | **X** |  | **X** | **X** |  |  |  | **X** |  |
| **ADOS-2^c^** | **X** |  |  |  |  |  |  |  |  |  |  |  |  |  |  |
| **DSM-5 Checklist** | **X** |  |  |  |  |  |  |  |  |  |  |  |  |  |  |
| **WASI-II** |  | **X** |  |  |  |  |  |  |  |  |  |  |  |  |  |
| **SCQ** |  | **X** |  |  |  |  |  |  |  |  |  |  |  |  |  |
| **CGI-S** |  | **X** |  |  |  |  | **X** |  | **X** |  |  |  |  | **X** |  |
| **CGI-I** |  |  |  |  |  |  | **X** |  |  |  |  |  |  | **X** |  |
| **Vineland-3** |  | **X** |  |  |  |  | **X** |  | **X** |  |  |  |  | **X** |  |

|  | **Day**  **-14 to 0** | **Day 1** | **Day 1** | **Day 7 (+/-2)** | **Day 14 (+/-2)** | **Day 21 (+/- 2)** | **Day 28 (+/-3)** | **Day 35**  **(+/-2)** | **Day 1^h^** | **Day 1** | **Day 7 (+/-2)** | **Day 14 (+/-2)** | **Day 21 (+/- 2)** | **Day 28 (+/-3)** | **Post-trial Washout** |
| --- | --- | --- | --- | --- | --- | --- | --- | --- | --- | --- | --- | --- | --- | --- | --- |
| **Measure** | **Screen** | **Visit 1: Pre-dose** | **Visit 1: Post-dose** | **Follow- Up 1**  **Phone** | **Follow -Up 2**  **Phone** | **Follow- Up 3**  **Phone** | **Visit 2: Final Visit of Period 1** | **Wash out period call** | **Visit 3: Pre-dose** | **Visit 3: Post-dose** | **Follow Up 1**  **Phone** | **Follow Up 2**  **Phone** | **Follow Up 3 Phone** | **Visit 4: End-of-Study/Early Termination Visit** | **Day 42 (+4)** |
| **ABC** |  | **X** |  |  |  |  | **X** |  | **X** |  |  |  |  | **X** |  |
| **ECG^d^** | **X** | **X** |  |  |  |  | **X** |  | **X** |  |  |  |  | **X** |  |
| **EEG/ERP/Chirp** |  | **X** |  |  |  |  | **X** |  | **X** |  |  |  |  | **X** |  |
| **Eye tracking** |  | **X** |  |  |  |  | **X** |  | **X** |  |  |  |  | **X** |  |
| **WJ3 Subtests** |  | **X** |  |  |  |  | **X** |  | **X** |  |  |  |  | **X** |  |
| **RBANS** |  | **X** |  |  |  |  | **X** |  | **X** |  |  |  |  | **X** |  |
| **KiTap** |  | **X** |  |  |  |  | **X** |  | **X** |  |  |  |  | **X** |  |
| **IP Compliance** |  |  |  | **X** | **X** | **X** | **X** |  |  |  | **X** | **X** | **X** | **X** |  |
| **AE Review** |  |  | **X** | **X** | **X** | **X** | **X** | **X** | **X** | **X** | **X** | **X** | **X** | **X** | **X** |
| **Concomitant Medication Review** | **X** | **X** |  | **X** | **X** | **X** | **X** | **X** | **X** |  | **X** | **X** | **X** | **X** |  |
| **Stool Sample** |  | **X^e^** |  |  |  |  | **X** | **X^f^** |  |  |  |  |  | **X** | **X^g^** |

1. A full physical exam will be done at screening. A limited focused physical exam may be done in case of adverse events at all other visits, as determined by the investigator
2. Safety labs will include hematology, chemistry and urinalysis
3. ADOS 2 results can be obtained from the medical record and used for the study if completed within the previous 36 months. If results for the ADOS are available, it will not be completed.
4. ECG will be done at either the screening or baseline visit for participants with AD.
5. This is a pretreatment stool sample, to be collected within approximately 48 hours before receiving the first dose of the IP.
6. Should be taken approximately 7 days into the wash-out period after the first treatment period of the study and before the second treatment period
7. This is post treatment stool sample, to be collected approximately 7 days after the completion of the second treatment period of the study
8. Study Period 2 Begins on Day 42 of the study or up to 7 days later

ADOS-2= Autism Diagnostic Observation Schedule Modules 2, 3 or 4; WASI-II= Wechsler Abbreviated Scale of Intelligence Scale-Second Edition, SCQ= social communication questionnaire; CGI-S= clinical global impressions severity scale, CGI-I=clinical global impressions improvement scale; Vineland-3= Vinland Adaptive Behaviors Scales 3^rd^ edition; PK= pharmacokinetics; EEG= electroencephalogram protocol; WJ3= Woodcock Johnson Spatial Relations and Auditory Attention subtests; RBANS= repeatable battery of neuropsychological status; KiTap= computerized test of attentional performance in children; ABC= aberrant behavior checklist;

**Table S3: Summary of Concomitant Medications – Period 1**

| Treatment Period   ATC Group  Preferred Term [a] |  | SB-121  (N=7)  n(%) | | | Placebo  (N=8)  n(%) | | Total  (N=15)  n(%) | |
| --- | --- | --- | --- | --- | --- | --- | --- | --- |
| Treatment Period 1, n |  | 7 | | | 8 | | 15 | |
| Number of Participants with any concomitant medication |  | 7 (100.0) | | | 8 (100.0) | | 15 (100.0) | |
| NERVOUS SYSTEM |  | 6 ( 85.7) | | | 8 (100.0) | | 14 ( 93.3) | |
| Melatonin |  | 3 ( 42.9) | | | 1 ( 12.5) | | 4 ( 26.7) | |
| Acamprosate |  | 1 ( 14.3) | | | 2 ( 25.0) | | 3 ( 20.0) | |
| Buspirone |  | 1 ( 14.3) | | | 2 ( 25.0) | | 3 ( 20.0) | |
| Clonidine |  | 1 ( 14.3) | | | 2 ( 25.0) | | 3 ( 20.0) | |
| Dexmethylphenidate hydrochloride |  | 1 ( 14.3) | | | 2 ( 25.0) | | 3 ( 20.0) | |
| Lisdexamfetamine mesylate |  | 1 ( 14.3) | | | 2 ( 25.0) | | 3 ( 20.0) | |
| Quetiapine |  | 1 ( 14.3) | | | 2 ( 25.0) | | 3 ( 20.0) | |
| Sertraline |  | 1 ( 14.3) | | | 2 ( 25.0) | | 3 ( 20.0) | |
| Amfetamine aspartate;Amfetamine sulfate  Sulfate;Dexamfetamine  Saccharate; Dexamfetamine Sulfate |  | 1 ( 14.3) | | | 1 ( 12.5) | | 2 ( 13.3) | |
| Guanfacine hydrochloride |  | 1 ( 14.3) | | | 1 ( 12.5) | | 2 ( 13.3) | |
| Methylphenidate hydrochloride |  | 2 ( 28.6) | | | 0 | | 2 ( 13.3) | |
| Risperidone |  | 1 ( 14.3) | | | 1 ( 12.5) | | 2 ( 13.3) | |
| Aripiprazole |  | 0 | | | 1 ( 12.5) | | 1 ( 6.7) | |
| Bupropion | | |  | 1 ( 14.3) | | 0 | | 1 ( 6.7) |
| Citalopram | | |  | 0 | | 1 ( 12.5) | | 1 ( 6.7) |
| Clonazepam | | |  | 0 | | 1 ( 12.5) | | 1 ( 6.7) |
| Dextromethorphan hydrobromide;Doxylamine  succinate;Ephedrine sulfate;Ethanol;Paracetamol | | |  | 0 | | 1 ( 12.5) | | 1 ( 6.7) |
| Dextromethorphan hydrobromide;Guaifenesin;  Paracetamol;Pseudoephedrine hydrochloride | | |  | 0 | | 1 ( 12.5) | | 1 ( 6.7) |
| Fluoxetine | | |  | 0 | | 1 ( 12.5) | | 1 ( 6.7) |
| Gabapentin | | |  | 0 | | 1 ( 12.5) | | 1 ( 6.7) |
| Guanfacine | | |  | 0 | | 1 ( 12.5) | | 1 ( 6.7) |
| Propranolol | | |  | 0 | | 1 ( 12.5) | | 1 ( 6.7) |
| ALIMENTARY TRACT AND METABOLISM |  | 4 ( 57.1) | | | 4 ( 50.0) | | 8 ( 53.3) | |
| Metformin |  | 1 ( 14.3) | | | 2 ( 25.0) | | 3 ( 20.0) | |
| Vitamin D NOS |  | 2 ( 28.6) | | | 1 ( 12.5) | | 3 ( 20.0) | |
| Vitamins NOS |  | 1 ( 14.3) | | | 1 ( 12.5) | | 2 ( 13.3) | |
| Alpha-amylase swine pancrease;cellulase;lipase;protease  NOS |  | 0 | | | 1 ( 12.5) | | 1 ( 6.7) | |
| Ascorbic acide |  | 0 | | | 1 ( 12.5) | | 1 ( 6.7) | |
| Calcium;Magnesium;Vitamin D NOS |  | 1 ( 14.3) | | | 0 | | 1 ( 6.7) | |
| Colecalciferol |  | 0 | | | 1 ( 12.5) | | 1 ( 6.7) | |
| Dicycloverine hydrochloride |  | 0 | | | 1 ( 12.5) | | 1 ( 6.7) | |
| Macrogol 3350 |  | 0 | | | 1 ( 12.5) | | 1 ( 6.7) | |
| Magnesium |  | 1 ( 14.3) | | | 0 | | 1 ( 6.7) | |
| Vitamin B complex |  | 0 | | | 1 ( 12.5) | | 1 ( 6.7) | |
| VARIOUS |  | 2 ( 28.6) | | | 1 ( 12.5) | | 3 ( 20.0) | |
| Fish oil |  | 2 ( 28.6) | | | 0 | | 2 ( 13.3) | |
| Omega-3 NOS |  | 0 | | | 1 ( 12.5) | | 1 ( 6.7) | |
| ANTIINFECTIVES FOR SYSTEMIC USE |  | 2 ( 28.6) | | | 0 | | 2 ( 13.3) | |
| Amoxicillin;Clavulanic acid |  | 1 ( 14.3) | | | 0 | | 1 ( 6.7) | |
| Azithromycin |  | 1 ( 14.3) | | | 0 | | 1 ( 6.7) | |
| Valaciclovir hydrochloride |  | 1 ( 14.3) | | | 0 | | 1 ( 6.7) | |
| MUSCULO-SKELETAL SYSTEM |  | 0 | | | 2 ( 25.0) | | 2 ( 13.3) | |
| Diphenhydramine citrate;Ibuprofen |  | 0 | | | 1 ( 12.5) | | 1 ( 6.7) | |
| Ibuprofen |  | 0 | | | 1 ( 12.5) | | 1 ( 6.7) | |
| CARDIOVASCULAR SYSTEM |  | 1 ( 14.3) | | | 0 | | 1 ( 6.7) | |
| Epinephrine |  | 1 ( 14.3) | | | 0 | | 1 ( 6.7) | |
| DERMATOLOGICALS |  | 1 ( 14.3) | | | 0 | | 1 ( 6.7) | |
| Mineral oil light;Paraffin;Petrolatum;Wool alcohols |  | 1 ( 14.3) | | | 0 | | 1 ( 6.7) | |
| RESPIRATORY SYSTEM |  | 1 ( 14.3) | | | 0 | | 1 ( 6.7) | |
| Cetirizine hydrochloride |  | 1 ( 14.3) | | | 0 | | 1 ( 6.7) | |
| Fluticasone propionate |  | 1 ( 14.3) | | | 0 | | 1 ( 6.7) | |
| Montelukast sodium |  | 1 ( 14.3) | | | 0 | | 1 ( 6.7) | |
| SYSTEMIC HORMONAL PREPARATIONS, EXCL. SEX HORMONES AND INSULINS |  | 1 ( 14.3) | | | 0 | | 1 ( 6.7) | |
| Prednisone |  | 1 ( 14.3) | | | 0 | | 1 ( 6.7) | |

Abbreviations: N = number of subjects according to the first treatment sequence; n = number of subjects with data available; ATC = Anatomical Therapeutic Chemical. % = percentages are calculated based on N as the denominator. Notes: Prior medications are those that started prior to start of dosing in Treatment Period 1. Prior medications were coded using WHODrug Global, Format B3, Version September 2021 [a] Within a Anatomical Group, a subject may have reported more than one preferred term. Subjects are counted once for each preferred term and each Anatomical Group.

**Table S4: Summary of Concomitant Medications – Period 2**

| Treatment Period   ATC Group  Preferred Term [a] |  | SB-121  (N=7)  n(%) | Placebo  (N=8)  n(%) | Total  (N=15)  n(%) |
| --- | --- | --- | --- | --- |
| Treatment Period 2, n |  | 8 | 7 | 15 |
| Number of Participants with any concomitant medication |  | 7 ( 87.5) | 7 (100.0) | 14 ( 93.3) |
| NERVOUS SYSTEM |  | 7 ( 87.5) | 6 ( 85.7) | 13 ( 86.7) |
| Melatonin |  | 1 ( 12.5) | 3 ( 42.9) | 4 ( 26.7) |
| Acamprosate |  | 2 ( 25.0) | 1 ( 14.3) | 3 ( 20.0) |
| Buspirone |  | 2 ( 25.0) | 1 ( 14.3) | 3 ( 20.0) |
| Clonidine |  | 2 ( 25.0) | 1 ( 14.3) | 3 ( 20.0) |
| Dexmethylphenidate hydrochloride |  | 2 ( 25.0) | 1 ( 14.3) | 3 ( 20.0) |
| Lisdexamfetamine mesylate |  | 2 ( 25.0) | 1 ( 14.3) | 3 ( 20.0) |
| Quetiapine |  | 2 ( 25.0) | 1 ( 14.3) | 3 ( 20.0) |
| Sertraline |  | 2 ( 25.0) | 1 ( 14.3) | 3 ( 20.0) |
| Amfetamine aspartate;Amfetamine sulfate  Sulfate;Dexamfetamine  Saccharate; Dexamfetamine Sulfate |  | 1 ( 12.5) | 1 ( 14.3) | 2 ( 13.3) |
| Guanfacine hydrochloride |  | 1 ( 12.5) | 1 ( 14.3) | 2 ( 13.3) |
| Methylphenidate hydrochloride |  | 0 | 2 ( 28.6) | 2 ( 13.3) |
| Risperidone |  | 1 ( 12.5) | 1 ( 14.3) | 2 ( 13.3) |
| Aripiprazole |  | 1 ( 12.5) | 0 | 1 ( 6.7) |
| Bupropion |  | 0 | 1 ( 14.3) | 1 ( 6.7) |
| Citalopram |  | 1 ( 12.5) | 0 | 1 ( 6.7) |
| Clonazepam |  | 1 ( 12.5) | 0 | 1 ( 6.7) |
| Fluoxetine |  | 1 ( 12.5) | 0 | 1 ( 6.7) |
| Gabapentin |  | 1 ( 12.5) | 0 | 1 ( 6.7) |
| Guanfacine |  | 1 ( 12.5) | 0 | 1 ( 6.7) |
| Propranolol |  | 1 ( 12.5) | 0 | 1 ( 6.7) |
| ALIMENTARY TRACT AND METABOLISM |  | 4 ( 50.0) | 4 ( 57.1) | 8 ( 53.3) |
| Metformin |  | 2 ( 25.0) | 1 ( 14.3) | 3 ( 20.0) |
| Vitamin D NOS |  | 1 ( 12.5) | 2 ( 28.6) | 3 ( 20.0) |
| Vitamins NOS |  | 1 ( 12.5) | 1 ( 14.3) | 2 ( 13.3) |
| Alpha-amylase swine pancrease;cellulase;lipase;protease  NOS |  | 1 ( 12.5) | 0 | 1 ( 6.7) |
| Ascorbic acide |  | 1 ( 12.5) | 0 | 1 ( 6.7) |
| Calcium;Magnesium;Vitamin D NOS |  | 0 | 1 ( 14.3) | 1 ( 6.7) |
| Colecalciferol |  | 1 ( 12.5) | 0 | 1 ( 6.7) |
| Dicycloverine hydrochloride |  | 1 ( 12.5) | 0 | 1 ( 6.7) |
| Macrogol 3350 |  | 1 ( 12.5) | 0 | 1 ( 6.7) |
| Magnesium |  | 0 | 1 ( 14.3) | 1 ( 6.7) |
| Vitamin B complex |  | 1 ( 12.5) | 0 | 1 ( 6.7) |
| VARIOUS |  | 1 ( 12.5) | 2 ( 28.6) | 3 ( 20.0) |
| Fish oil |  | 0 | 2 ( 28.6) | 2 ( 13.3) |
| Omega-3 NOS |  | 1 ( 12.5) | 0 | 1 ( 6.7) |
| ANTIINFECTIVES FOR SYSTEMIC USE |  | 0 | 2 ( 28.6) | 2 ( 13.3) |
| Influenza vaccine |  | 0 | 1 ( 14.3) | 1 ( 6.7) |
| Valaciclovir hydrochloride |  | 0 | 1 ( 14.3) | 1 ( 6.7) |
| CARDIOVASCULAR SYSTEM |  | 0 | 1 ( 14.3) | 1 ( 6.7) |
| Epinephrine |  | 0 | 1 ( 14.3) | 1 ( 6.7) |
| DERMATOLOGICALS |  | 0 | 1 ( 14.3) | 1 ( 6.7) |
| Ketoconazole |  | 0 | 1 ( 14.3) | 1 ( 6.7) |
| Mineral oil light;Paraffin;Petrolatum;Wool alcohols |  | 0 | 1 ( 14.3) | 1 ( 6.7) |
| Terbinafine |  | 0 | 1 ( 14.3) | 1 ( 6.7) |
| MUSCULO-SKELETAL SYSTEM |  | 1 ( 12.5) | 0 | 1 ( 6.7) |
| Ibuprofen |  | 1 ( 12.5) | 0 | 1 ( 6.7) |
| RESPIRATORY SYSTEM |  | 0 | 1 ( 14.3) | 1 ( 6.7) |
| Cetirizine hydrochloride |  | 0 | 1 ( 14.3) | 1 ( 6.7) |
| Fluticasone propionate |  | 0 | 1 ( 14.3) | 1 ( 6.7) |
| Montelukast sodium |  | 0 | 1 ( 14.3) | 1 ( 6.7) |

Abbreviations: N = number of subjects according to the first treatment sequence; n = number of subjects with data available; ATC = Anatomical Therapeutic Chemical. % = percentages are calculated based on N as the denominator. Notes: Prior medications are those that started prior to start of dosing in Treatment Period 1. Prior medications were coded using WHODrug Global, Format B3, Version September 2021 [a] Within a Anatomical Group, a subject may have reported more than one preferred term. Subjects are counted once for each preferred term and each Anatomical Group.

**Table S5: Individual Vineland Adaptive Behavior Scale Scores (change from baseline).**

| **Subject ID** | **Vineland Adaptive Behavior Composite Score** | | | **Vineland Socialization Domain** | | | **Vineland Daily Living Skills Domain** | | | **Vineland Communication Domain** | | |
| --- | --- | --- | --- | --- | --- | --- | --- | --- | --- | --- | --- | --- |
|  | **SB-121** | **Placebo** | **Δ^[[1]](#footnote-1)^** | **SB-121** | **Placebo** | **Δ^b^** | **SB-121** | **Placebo** | **Δ^b^** | **SB-121** | **Placebo** | **Δ^b^** |
| **001** | 12 | -12 | 24 | 24 | -21 | 45 | 8 | 0 | 8 | 4 | -12 | 16 |
| **002** | 13 | 4 | 9 | 9 | -2 | 11 | 8 | 20 | -12 | 24 | -8 | 32 |
| **003** | -4 | 11 | -15 | 4 | 9 | -5 | -8 | 0 | -8 | -8 | 28 | -36 |
| **004** | 13 | -6 | 19 | 23 | -5 | 28 | 17 | -11 | 28 | 4 | -6 | 10 |
| **005** | 6 | 2 | 4 | 3 | -8 | 11 | 18 | 11 | 7 | -3 | 4 | -7 |
| **006** | 0 | 3 | -3 | -14 | 5 | -19 | 4 | -5 | 9 | 10 | 7 | 3 |
| **008** | 9 | -1 | 10 | 10 | 2 | 8 | 3 | -4 | 7 | 15 | -1 | 16 |
| **009** | 22 | 1 | 21 | 2 | -6 | 8 | 27 | 6 | 21 | 30 | 2 | 28 |
| **010** | -3 | -8 | 5 | -1 | 0 | -1 | 3 | -6 | 9 | -13 | -21 | 8 |
| **011** | 3 | 8 | -5 | 3 | 18 | -15 | 3 | 6 | -3 | 2 | 0 | 2 |
| **012** | 18 | 6 | 12 | 26 | -4 | 30 | 0 | -13 | 13 | 18 | 28 | -10 |
| **013** | -6 | -5 | -1 | -6 | 6 | -12 | 2 | -6 | 8 | -14 | -16 | 2 |
| **014** | -2 | 0 | -2 | -13 | 5 | -18 | 5 | -2 | 7 | 0 | -2 | 2 |
| **015** | -7 | 4 | -11 | 4 | -6 | 10 | -8 | 6 | -14 | -23 | 14 | -37 |
| **016** | 0 | 12 | -12 | 8 | 8 | 0 | -2 | 8 | -10 | -6 | 27 | -33 |

Participants 001, 002, 004, 008, 009 and 012 were the 6 participants with a placebo adjusted change from baseline of ≥8 on SB-121 on the Adaptive Behavior Composite score.

b SB-121 minus Placebo.

Table S6: Pre- and Post-Dose Data for CGI Scales

|  | **Placebo** | | | | **SB-121** | | | |
| --- | --- | --- | --- | --- | --- | --- | --- | --- |
|  | **Pre-Dose** | | **Post-Dose** | | **Pre-Dose** | | **Post-Dose** | |
|  | **Mean** | **SEM** | **Mean** | **SEM** | **Mean** | **SEM** | **Mean** | **SEM** |
| Clinical Global Impressions Severity Subscale | 3.87 | 0.13 | 3.93 | 0.12 | 3.93 | 0.12 | 3.87 | 0.13 |
| Clinical Global Impressions Improvement Subscale | NA | NA | 3.6 | 0.16 | NA | NA | 3.47 | 0.26 |

**Table S7: Summary of Overall Plasma Oxytocin Levels**

|  | SB-121 | | | Placebo | | |
| --- | --- | --- | --- | --- | --- | --- |
|  | Value at Visit | Change from  Baseline | Percentage Change from Baseline | Value at Visit | Change from Baseline | Percentage Change from Baseline |
| Oxytocin (pg/mL) | | | | | | |
| Baseline |  |  |  |  |  |  |
| n | 15 |  |  | 15 |  |  |
| Mean (SD) | 37.0647  (23.54037) |  |  | 41.2383  (22.70178) |  |  |
| Median | 27.0600 |  |  | 34.5700 |  |  |
| Min, Max | 15.625,  74.690 |  |  | 15.625,  101.880 |  |  |
| End of Treatment |  |  |  |  |  |  |
| n | 14 | 14 | 14 | 13 | 13 | 13 |
| Mean (SD) | 53.3025  (23.05427) | 17.9539  (30.42421) | 111.6278  (155.93306) | 44.2181  (18.15442) | 0.8515  (22.75805) | 24.6667  (80.94121) |
| Median | 56.6650 | 17.4525 | 55.5703 | 45.9900 | 1.6500 | 3.6748 |
| Min, Max | 15.625,  103.930 | -31.290,  84.860 | -42.258,  444.992 | 15.625,  71.260 | -43.660,  37.390 | -44.142,  191.842 |

Abbreviations; n = number of participants with data available; SD = Standard deviation; Min = Minimum; Max = Maximum; LLQ = Lower limit quantification.

1. [↑](#footnote-ref-1)
